# Microbiota transplants: the concept of ‘microbiome mismatching’ explored

**DOI:** 10.1038/s41392-025-02464-9

**Published:** 2025-11-17

**Authors:** Benjamin H. Mullish, Lauren A. Roberts, Horace R. T. Williams

**Affiliations:** 1 Division of Digestive Diseases, Department of Metabolism, Digestion and Reproduction, Faculty of Medicine, Imperial College London, London, UK; 2 Departments of Gastroenterology and Hepatology, St Mary’s Hospital, Imperial College Healthcare NHS Trust, London, UK

**Keywords:** Microbiology, Preclinical research

Research highlight related to: DeLeon O., Mocanu M., Tan A., Sidebottom A.M., Koval J. et al. Cell (2025);188, 3927-3941, 10.1016/j.cell.2025.05.014.

In a recent paper published in *Cell*,^1^ DeLeon and colleagues compared the impact of faecal microbiota transplantation (FMT) with the outcomes of transplanting jejunal or caecal microbiota material, to explore the concept of ‘microbiome matching/ mismatching’. They observed differential impacts upon host-microbe interactions based on the source of the microbiota, and expressed concern about ‘unintended consequences’ of microbial therapeutics in clinical practice.

FMT has a well-established role in treating recurrent *Clostridioides difficile* infection (CDI), and shows therapeutic promise in numerous non-CDI settings.^2^ While the efficacy of FMT relates at least partly to engraftment of the donor microbiome within the recipient’s microbiome, the degree to which engraftment correlates with success of FMT remains debated.^3^ Mechanistic mediators of the efficacy of FMT are also incompletely understood, although these appear to include alterations to gut microbial metabolites and host immune response.^3^ Consequently, factors which might influence FMT engraftment and its potential alteration of host physiology are of interest, as such variables may impact upon clinical outcomes. The authors here explored one such factor - the intestinal site from which donor material is sourced, and its ‘matching’ with the site reached in the recipient.

The hypothesis was based on recognition of the profound differences in composition and functionality of gut microbial ecosystems between small and large bowel, adapted to the different host physiological functions of these sites. The authors made a clinical observation of the presence of strict anaerobes within the small intestinal content of seven human patients one month post-FMT (based on microbiome sequencing), highlighting the potential for colonisation of colonically-derived bacteria within the small bowel microbiome, and raising questions about implications of this ‘mismatch’.

To investigate further, specific pathogen free (SPF) mice were treated with antibiotics, before a single per-oral treatment with either jejunal, caecal or faecal microbiota transplant (JMT/ CMT/ FMT), with sample analysis performed up to three months post-treatment. Regional microbiome composition reflected both the site of donor microbiota collection and the recipient gastrointestinal (GI) tract region being sampled, with beta diversity separating both based on a small or large intestinal source of donor microbiota, and similarly on whether recipient microbiota sampling related to the small intestine or colon. JMT colonised the aerobic jejunum best, while CMT and FMT optimally colonised the anaerobic caecum and colon, although all sources of microbiota transplant colonised the whole of the intestinal tract to some degree. Metabolomic analyses of mouse intestinal content (from both colon and duodenum/ jejunum) and plasma similarly demonstrated differential changes to a range of metabolites that are influenced by both microbiome and host (including short chain fatty acids and bile acids), related to whether the source the microbiome transplant was small or large intestinal in origin. Replication of these experiments using germ-free mice showed broadly comparable results.

Since gut microbial metabolites are transported from the gut to the liver via the portal vein, the impact of the source of microbiota transplant on hepatic function was explored. Liver transcriptomes of transplanted mice differed particularly between JMT and FMT recipients; JMT-treated mice demonstrated enrichment in metabolic-associated genes, with FMT-treated mice enriched in immune-related genes. Differences in metabolic phenotype were also seen, with JMT-treated mice having lower energy expenditure compared to FMT-treated. Transcriptional analysis of mouse intestinal mucosa demonstrated that transcriptomes of key cell differential regulators (including *Gata4* and *Gata6* for jejunum, and *Satb2* for colon) were expressed at higher levels in tissue from mice receiving matched microbiota transplants compared to those with mismatches.

To further explore potential relevance to humans, the authors exposed cultured primary human jejunal enteroids from eight people to a 10% acellular preparation of human jejunal contents from a single jejunostomy donor, or to faecal slurry. Jejunal transcriptional changes differed between jejunal or faecal slurry exposure, with JMT-treated enteroids again showing enrichment in lipid biosynthesis genes.

The authors concluded that their data raise important questions about potential ‘off-target’ effects related to mismatched microbiota transplants, particularly where FMT is being delivered orally (which is increasingly the case clinically, in an era of emergent capsulised FMT). While these results may have potential implications for clinical practice, it is also appropriate to strike a degree of caution in interpreting these findings. Regarding the human studies, fewer than 10 patients provided samples for both the post-FMT and jejunal enteroid analyses, with single post-transplant timepoints examined in both scenarios, limiting assessment of the dynamic longer-term changes in host-microbiome interactions post-intervention. The presence of strict anaerobes in the small intestine post-FMT, and the microbiome-related changes in post-transplant host intestinal transcriptomes, is noteworthy, but the durability and practical significance of these changes is unclear. It is also uncertain whether this translates into any adverse metabolic or immunological clinical outcome, as inferred; overall, FMT appears a safe treatment, without any changes to such phenotypes observed in clinical cohorts with longer follow-up.^4^

A further caution concerns the translational significance of the mouse studies to humans. For instance, relatively modest changes in metabolomic and liver transcriptional changes were noted between SPF mice treated with FMT and saline alone, which was partly attributed to murine coprophagia and ongoing seeding of colonic microbiota into the upper gastrointestinal tract. This is in contrast to FMT in humans, which exerts profound alterations in intestinal metabolomic profiles.^5^ While the authors also performed microbiota transplant experiments in germ-free mice to mitigate this drawback, such mice are of limited biological comparability to a human patient, even those exposed to antibiotics due to recurrent CDI. Furthermore, the marked changes in intestinal transcriptomes between mice receiving different microbiota transplants were not fully replicated in human patients who received peroral FMT, raising further questions about translatability.

**Fig. 1:**
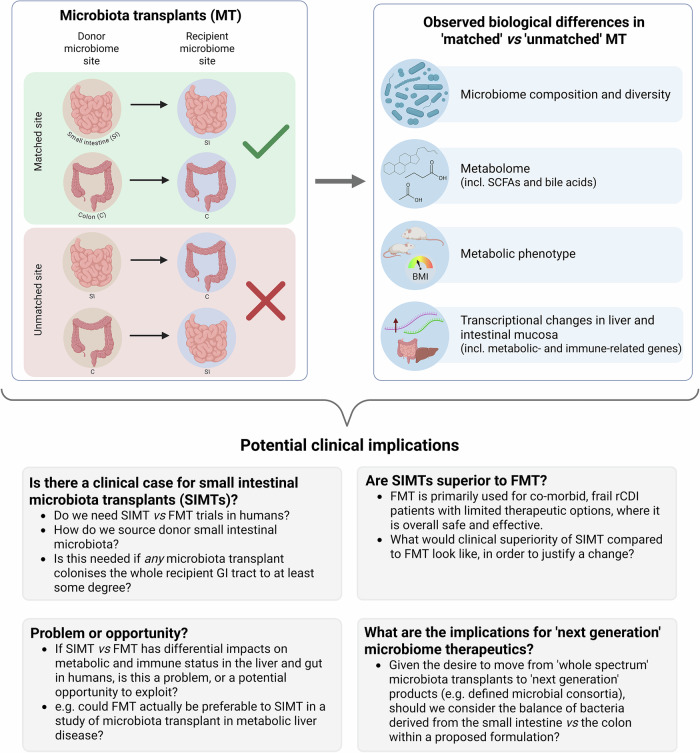
Summary of the main experimental findings of the paper from DeLeon and colleagues,^1^ and potential clinical implications: This study raises prompts discussion of a range of issues related to the use of microbiota transplants in humans, both from a clinical perspective, and from a research/ trial perspective. BMI body mass index, C colon, FMT faecal microbiota transplant, GI gastrointestinal, MT microbiota transplants, rCDI recurrent *Clostridioides difficile* infection, SCFAs short chain fatty acids, SI small intestine, SIMT small intestinal microbiota transplant. Figure created using BioRender

Despite these unanswered questions, this study raises thought-provoking questions that clinical practitioners of FMT should carefully consider (Fig. 1), and which will likely prompt further pre-clinical and clinical studies, particularly investigating the optimal source and composition of donor material. As ever, FMT as a human therapeutic should undoubtedly be used carefully, and according to the rigorous protocols detailed in national and international guidelines, with appropriate cautious recognition of the many outstanding ‘unknowns’ relating to its use.
